# Supplementary material for: Soluble guanylate cyclase signalling mediates etoposide resistance in progressing small cell lung cancer
Source: Nat Commun. 2021 Nov 17;12:6652. doi: 10.1038/s41467-021-26823-6 (PMC8599617; doi:10.1038/s41467-021-26823-6)
Supplement: Supplementary file 1 — Supplementary Information [file 41467_2021_26823_MOESM1_ESM.pdf]

Soluble Guanylate Cyclase Signalling Mediates Etoposide Resistance in Progressing Small Cell Lung Cancer

Maximilian W Schenk[1], Sam Humphrey[1], A S Md Mukarram Hossain[1], Mitchell Revill[1], Sarah Pearsall[1], Alice Lallo[1], Stewart Brown[1], Samuel Bratt[1], Melanie Galvin[1], Tine Descamps[1], Cong Zhou[1], Simon P Pearce[1], Lynsey Priest[1], Michelle Greenhalgh[2], Anshuman Chaturvedi[2], Alastair Kerr[1,3], Fiona Blackhall[2,3,4], Caroline Dive[1,3,*] and Kristopher K Frese[1,3,*].

[1]Cancer Research UK Manchester Institute Cancer Biomarker Centre, University of Manchester, Alderley Park, Macclesfield SK10 4TG, UK.

[2]Christie National Health Service Foundation Trust, Division of Cancer Sciences, The University of Manchester, Manchester M20 4BX, UK.

[3]Cancer Research UK Lung Cancer Centre of Excellence at the University of Manchester, Oxford Road, Manchester M13 9PL, UK.

[4]Division of Cancer Sciences, Faculty of Biology, Medicine and Health, University of Manchester, Manchester M13 9PL, UK.

*These authors jointly supervised this work. Correspondence: Caroline Dive, Cancer Research UK Manchester Institute Cancer Biomarker Centre, The University of Manchester, Alderley Park, SK10 4TG, UK, phone number: +44(0)161 306 6295, email: caroline.dive@manchester.ac.uk

Supplementary Figure 1

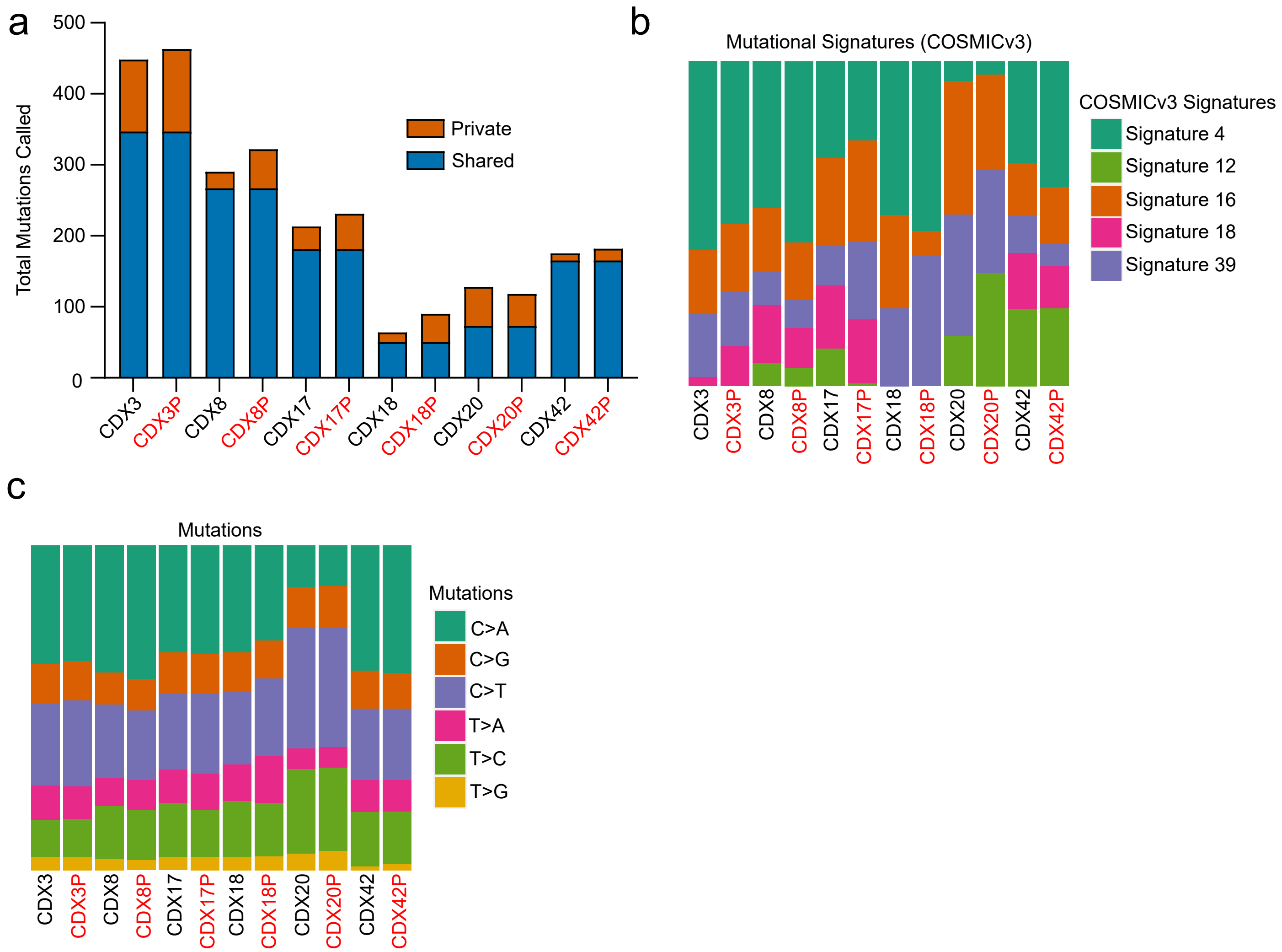

Supplementary Figure 1. Genomic Characterization of Clinical Resistance Models, Related to Figure 1

a) Comparison of genes affected by shared and private mutations in baseline and progression CDX models. There was a trend ($p = 0.06$, $t = -2.414$, $df = 5$) for an increased number of private mutations in progression models compared to baseline models. p values from two-sided paired Student's t test following Shapiro-Wilk test to confirm normality.

b) COSMIC 3 mutational signatures in baseline and progression CDX models for the top five signatures according to exposure. Signature 4 is associated with tobacco smoking.

c) Single base substitution profiles of baseline and progression CDX models.

Supplementary Figure 2

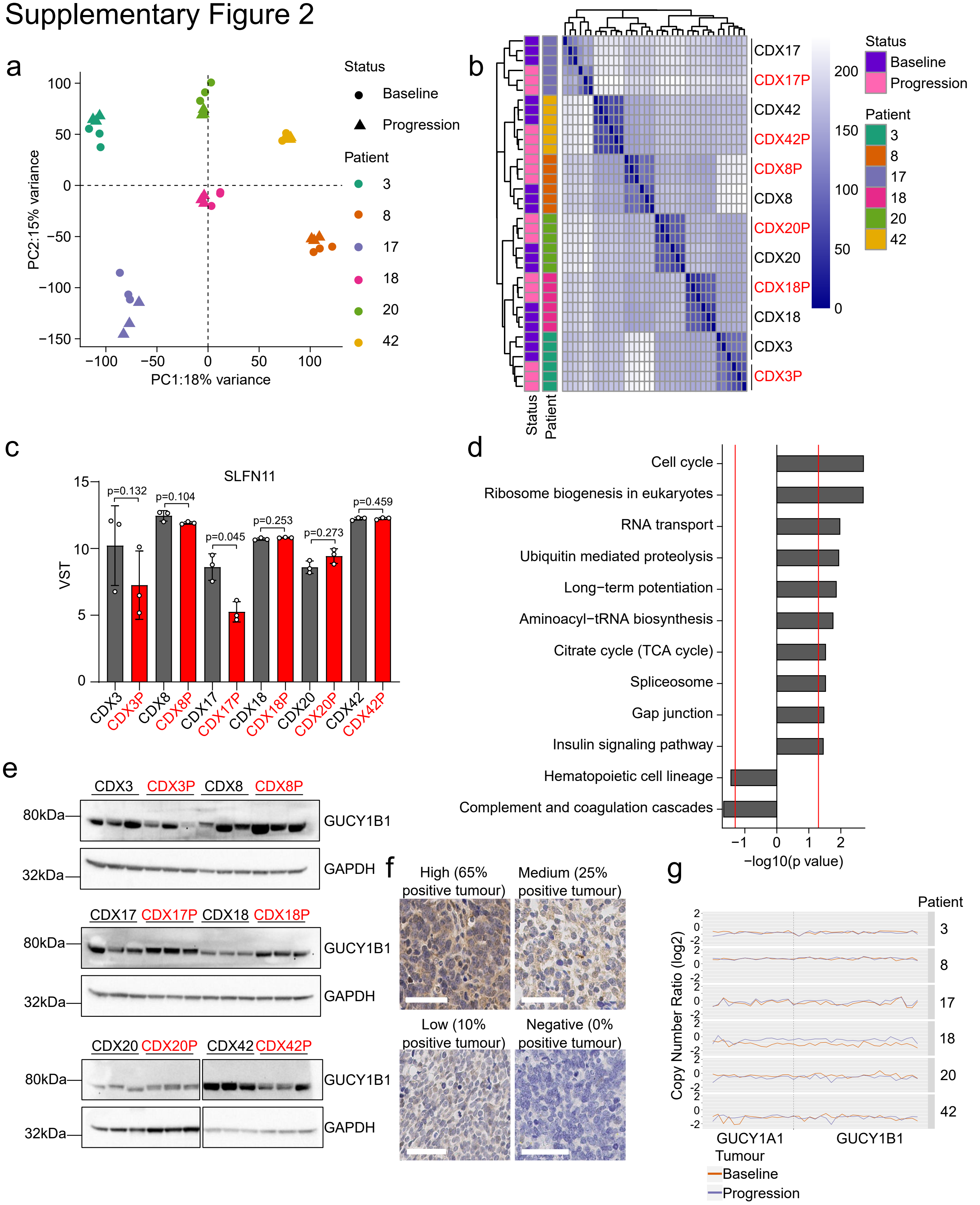

Supplementary Figure 2. sGCs are Upregulated During Disease Progression and Correlate with Acquired rather than Inherent Chemoresistance in SCLC, Related to Figure 2

a) Principal component analysis (PCA) of RNA-seq data from individual CDX progression models. Three replicates derived from three independent mice per model were used (n = 3).

b) Intersample similarity of RNA-seq data to show clustering of baseline and progression replicate samples. Three replicates derived from three independent mice per model were used (n = 3).

c) *SLFN11* expression (variance-stabilizing transformation, VST) of CDX progression models. At least three mice per cohort were used (n = 3). Data are represented as mean ± SD. p values from two-sided paired Student's t test.

d) Recurrently up- and downregulated pathways in CDX progression models. Pathway analysis using Generally Applicable Gene-set Enrichment (GAGE)[1], KEGG gene sets are displayed. Global p values were calculated by GAGE using a meta-test on the p-values[1].

e) Western blot for GUCY1B1 expression in paired pre-treatment and post-progression CDX tumour lysates. Western blot was performed on tumour lysates derived from three animals per model (n = 3).

f) Representative immunohistochemistry (IHC) staining for GUCY1B1 (% positive tumour) of SCLC tumour microarrays (TMAs) sourced through the approved Manchester Cancer Research Centre Biobank (MCRC). Scale bar set to 50 µm. Representative images of 3 independent replicates (n = 3) are shown.

g) Copy-number (CN) changes in GUCY1A1 and GUCY1B1 locus between baseline and progression CDX models calculated as log2 (copy number progression/copy number baseline). CN changes were estimated from the germline and tumour (baseline and progression) WES data.

Supplementary Figure 3

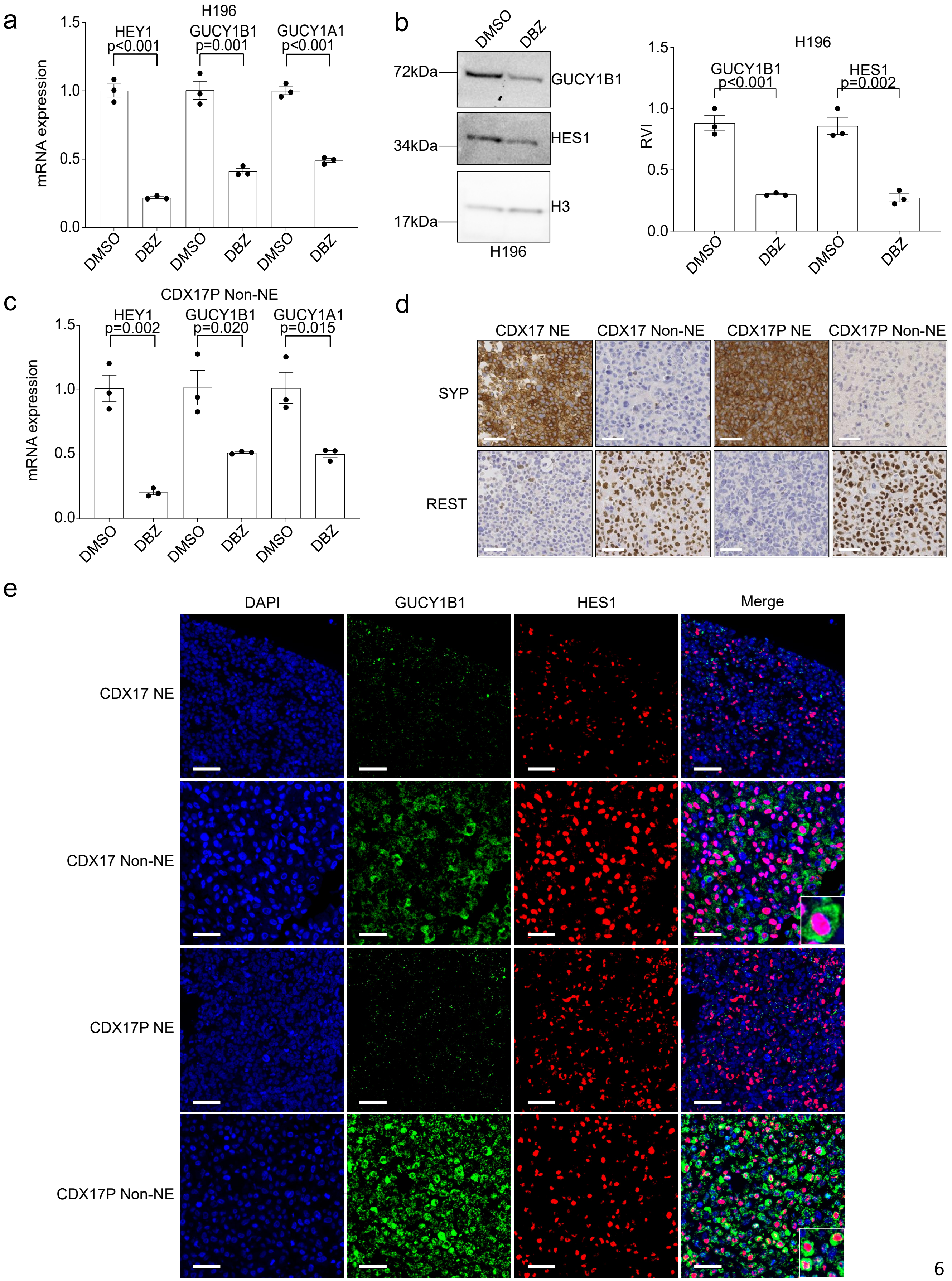

Supplementary Figure 3. Regulation of sGC Subunit Expression by Notch Signalling in SCLC, Related to Figure 3

a) *HEY1*, *GUCY1B1* and *GUCY1A1* mRNA expression measured by RT-qPCR of DMSO- or DBZ-treated H196 cells. Fold enrichment normalized to untreated control and Beta-2-Microglobulin *(B2M)* housekeeping gene expression. n = 3, data are represented as mean ± SEM. p values from two-sided unpaired Student's t test. p values: HEY1: 0.0001, GUCY1A1: 0.0001.

b) Western blot for GUCY1B1 and HES1 expression after treatment of H196 cells with DMSO or DBZ. Quantification of GUCY1B1 (relative volume intensity, RVI) on the right, normalized to H3 expression. n = 3, data are represented as mean ± SEM; p values from two-sided unpaired Student's t test. p value GUCY1B1: 0.0007.

c) *HEY1*, *GUCY1B1* and *GUCY1A1* mRNA expression measured by RT-qPCR of DMSO- or DBZ-treated CDX17P Non-NE cells. Fold enrichment normalized to untreated control and Beta-2-Microglobulin *(B2M)* housekeeping gene expression. n = 3, data are represented as mean ± SEM. p values from two-sided unpaired Student's t test.

d) IHC of CDX17 and CDX17P NE and Non-NE CDX cells for synaptophysin (SYP) and REST. Scale bar set to 50 µm and equivalent throughout panels.

e) Co-immunofluorescence for GUCY1B1 and HES1 in CDX17 and CDX17P NE and Non-NE CDX cells. Scale bar set to 50 µm and equivalent throughout panels. Insets show higher magnification images.

Supplementary Figure 4

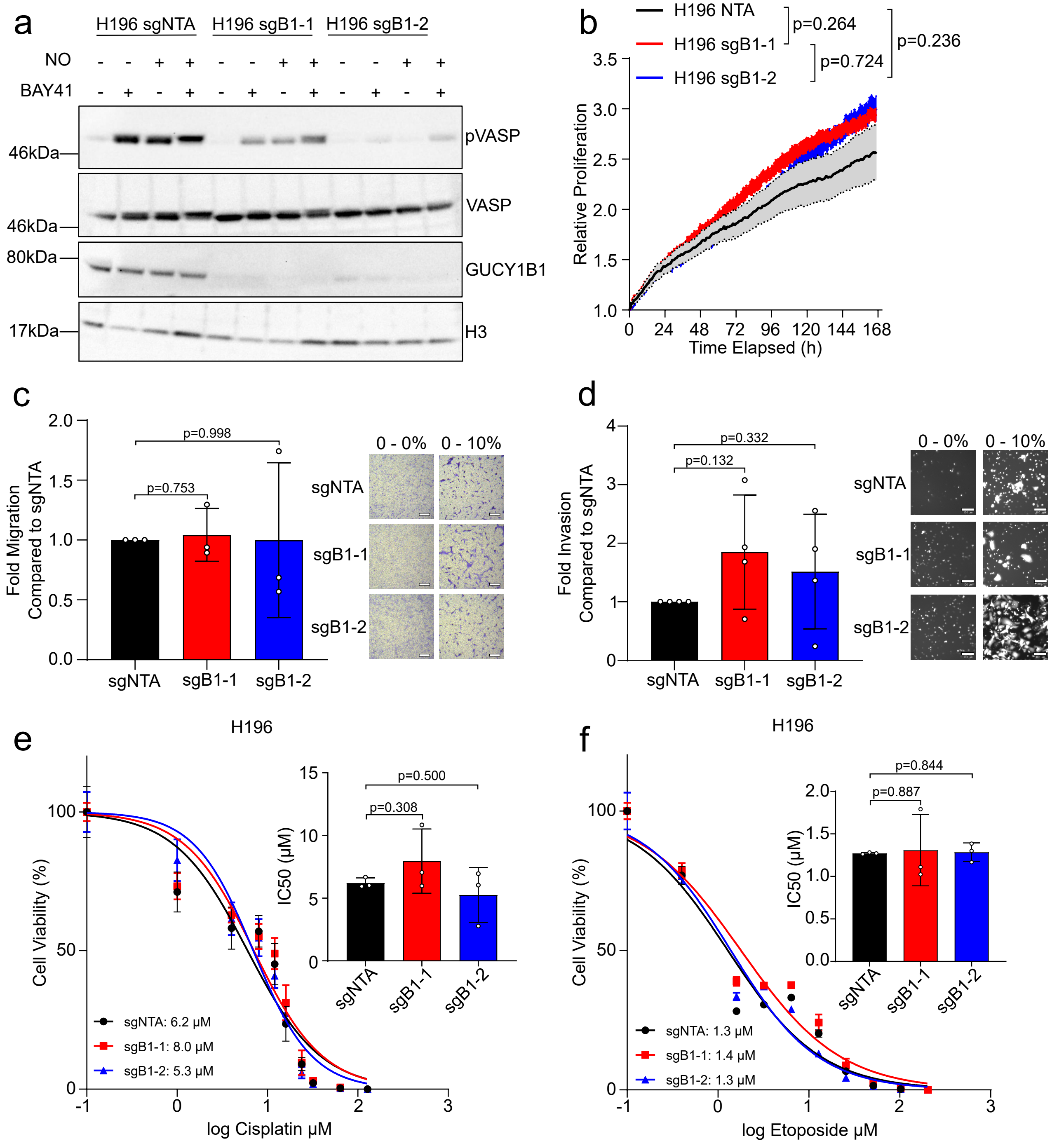

Supplementary Figure 4. Impact of sGC Signalling on SCLC Cell Fate and Chemotherapy Responses *in vitro*, Related to Figure 4

a) Western blot for pVASP, VASP, GUCY1B1 of untreated, BAY41, NO, and BAY41 + NO treated H196 sgNTA, sgB1-1, sgB1-2 cells. NO: nitric oxide, BAY41: sGC activator. n = 3.

b) IncuCyte proliferation assay of NO-treated H196 sgNTA, sgB1-1, sgB1-2 cells over a period of one week. n = 3, data are represented as mean ± SEM. p values from two-sided unpaired Student's t test.

c) Transwell migration assay of NO-treated H196 sgNTA, sgB1-1 and sgB1-2 cells migrating 24 hr towards a 10% serum gradient. n = 3, data are represented as mean ± SD. p values from two-sided unpaired Student's t test. Scale bar set to 50 μm and equivalent throughout panels.

d) Transwell invasion assay of NO-treated H196 sgNTA, sgB1-1, and sgB1-2 cells invading 24 hr through matrigel towards a 10% serum gradient. n = 4, data are represented as mean ± SD. p values from two-sided unpaired Student's t test. Scale bar set to 100 μm and equivalent throughout panels.

(e and f) Viability of H196 sgNTA, sgB1-1, and sgB1-2 cells treated with (e) cisplatin or (f) etoposide for one week. Representative data of three independent replicates are shown. Data as mean ± SD. IC_{50} values of three independent replicates are shown (n = 3), data are represented as mean ± SD. p values from two-sided unpaired Student's t test.

Supplementary Figure 5

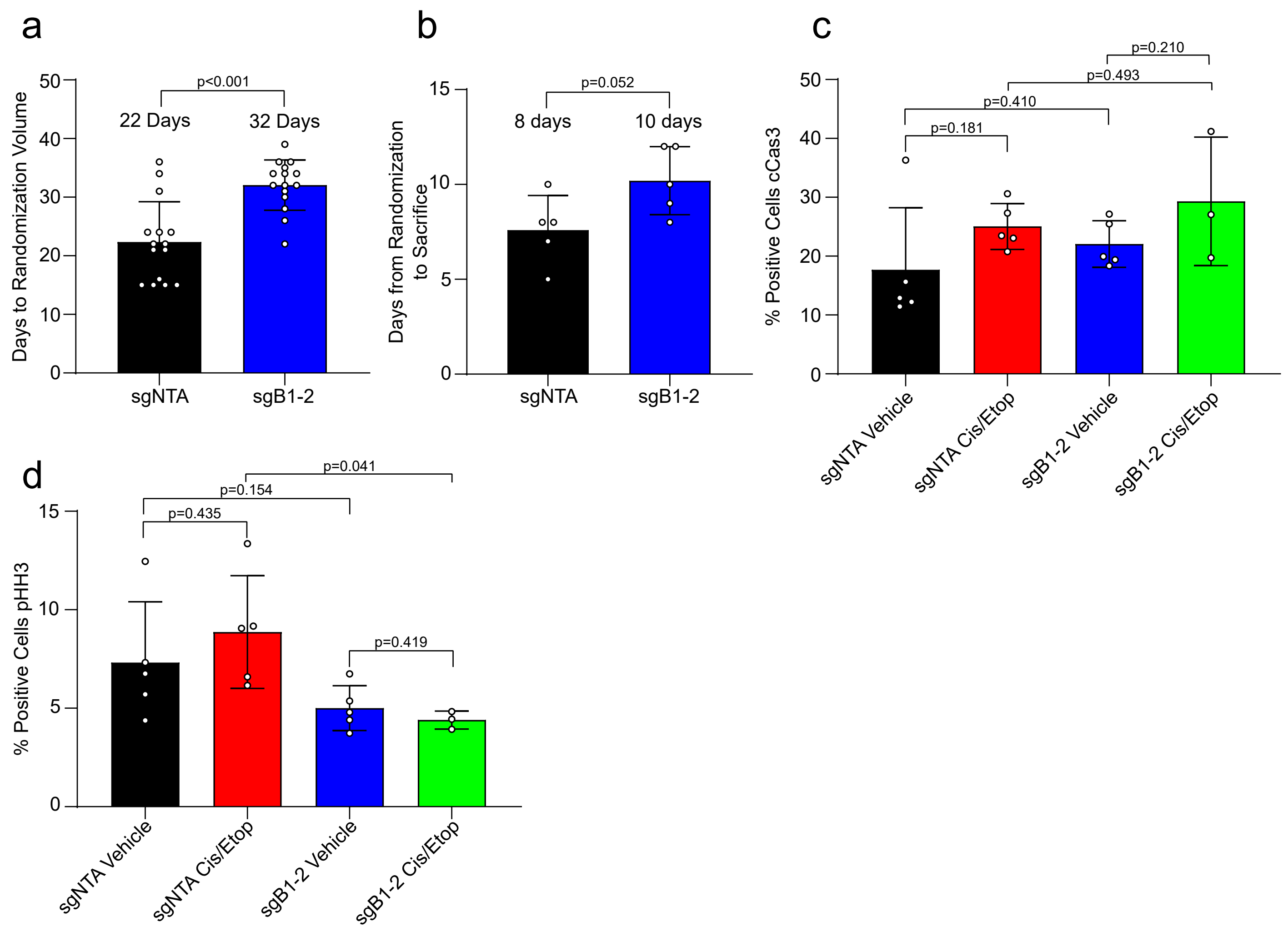

Supplementary Figure 5. Loss of sGC Signalling Sensitizes CDX17P to Cisplatin/Etoposide *in vivo*, Related to Figure 5

a) Days to randomization volume (200 mm^3) of mice implanted with CDX17P sgNTA or CDX17P sgB1-2 cells. 15 mice per group were analyzed (n = 15), data are represented as mean ± SD. p values from two-sided unpaired Student's t test. t = 4.64, df = 28. p value 0.0001.

b) Days from randomization volume (200 mm^3) to sacrifice of vehicle treated mice implanted with CDX17P sgNTA or CDX17P sgB1-2 cells. Five mice per group were analyzed (n = 5), data are represented as mean ± SD. p values from two-sided unpaired Student's t test. t = 2.28, df = 8.

(c and d) IHC for cleaved caspase-3 (cCas3) and phospho-histone H3 (pHH3) of CDX17P sgNTA and sgB1-2 tumours treated with vehicle or cisplatin/etoposide. Whole tumours were quantified using HALO software. For sgNTA vehicle, sgNTA cis/etop, and sgB1-2 vehicle cohorts, five tumours were quantified (n = 5), for sgB1-2 cis/etop cohort three tumours were quantified (n = 3), data are represented as mean ± SD. p values from two-sided unpaired Student's t test.

Supplementary Table 1. Genomic Characterization of Clinical Resistance Models, Related to Figure 1 and Supplementary Figure 1

Patient characteristics of CDX models used throughout the study. CR: Complete response, PR: Partial response, SR: Stable response, PD2: Progressive disease 2, PD1: Progressive disease 1. CDX response was evaluated using a preclinical RECIST score [2-4].

CDX Model	SCLC Subtype	Patient Diagnosis	Patient Metastasis	Patient Treatment	Patient Response	CDX Response (CDX/CDXP)	Patient Time to Progression
CDX3/ CDX3P	ASCL1	Extensive Stage SCLC	Nodes	Carboplatin/etoposide (2 cycles), radiotherapy	PR	CR/PR	207 days
CDX8/ CDX8P	NEUROD1	Extensive Stage SCLC	Bone, Liver	Single agent carboplatin (4 cycles)	PR	PR/PD1	57 days
CDX17/ CDX17P	ATOH1	Extensive Stage SCLC	Nodes, Liver	Carboplatin/etoposide (4 cycles), radiotherapy	PR	PD2/PD1	95 days
CDX18/ CDX18P	ASCL1	Extensive Stage SCLC	Liver, Adrenals, Bone	Carboplatin/etoposide (4 cycles), radiotherapy	PR	PD2/PD1	116 days
CDX20/ CDX20P	ASCL1	Extensive Stage SCLC	Nodes, Liver, Bone, Adrenals	Single agent carboplatin (1 cycle), radiotherapy	SR	PR/PD1	44 days
CDX42/ CDX42P	ASCL1	Extensive Stage SCLC	Bone, Pleural Effusion, Adrenals	Carboplatin/etoposide (3 cycles)	PR	CR/PR	59 days

Supplementary Table 2. List of Primers Used in This Study.

Name	Sequence
sgRNA Primers	
sgB1-1 Forward	CACCGGAGGCACAGTTAGATGAAGA
sgB1-1 Reverse	AAACTCTTCATCTAACTGTGCCTCC
sgB1-2 Forward	CACCGGAGAAATCCTCCAAATGTTT
sgB1-2 Reverse	AAACAAACATTTGGAGGATTTCTCC
sgNotch1-1 Forward	CACCGGAGGTGGCTGCGCAGCGACA
sgNotch1-1 Reverse	AAACTGTCGCTGCGCAGCCACCTCC
sgNotch1-2 Forward	CACCGGCATCTGTGCCAGTACGATG
sgNotch1-2 Reverse	AAACCATCGTACTGGCACAGATGCC
sgNTA Forward	CACCGCTGAAAAAGGAAGGAGTTGA
sgNTA Reverse	AAACTCAACTCCTTCCTTTTTCAGC
qPCR Primers ChIP	
HES1 Forward	CCTCCTCCCATTGGCTGAA
HES1 Reverse	CAGCTCCGGATCCTGTGTG
GUCY1A1 Forward	GGGACCAGATTAGATGGTGTACTTGG
GUCY1A1 Reverse	CCCATCATGCTGTTCCATTGTG
GUCY1B1 Forward	TTCATCATAGCTCTCTCGGTGCTG
GUCY1B1 Reverse	TGGACCTGAGAAGCCAGATGGTAT
Negative Control Forward	GCCCAGGGTTCTTGGAGAA
Negative Control Reverse	CATTCAGGCAGCGAGGACC

SUPPLEMENTARY REFERENCES

1 Luo, W., Friedman, M. S., Shedden, K., Hankenson, K. D. & Woolf, P. J. GAGE: generally applicable gene set enrichment for pathway analysis. *BMC Bioinformatics* **10**, 161, doi:10.1186/1471-2105-10-161 (2009).

2 Houghton, P. J. *et al.* The pediatric preclinical testing program: description of models and early testing results. *Pediatr Blood Cancer* **49**, 928-940, doi:10.1002/pbc.21078 (2007).

3 Geier, B., Kurmashev, D., Kurmasheva, R. T. & Houghton, P. J. Preclinical Childhood Sarcoma Models: Drug Efficacy Biomarker Identification and Validation. *Front Oncol* **5**, 193, doi:10.3389/fonc.2015.00193 (2015).

4 Simpson, K. L. *et al.* A biobank of small cell lung cancer CDX models elucidates inter- and intratumoral phenotypic heterogeneity. *Nature Cancer*, doi:10.1038/s43018-020-0046-2 (2020).
