# Supplementary material for: Soluble guanylate cyclase signalling mediates etoposide resistance in progressing small cell lung cancer
Source: Nat Commun. 2021 Nov 17;12:6652. doi: 10.1038/s41467-021-26823-6 (PMC8599617; doi:10.1038/s41467-021-26823-6)
Supplement: Supplementary file 3 — Description of Additional Supplementary Files [file 41467_2021_26823_MOESM3_ESM.docx]

Description of Additional Supplementary Files

Title: Supplementary Data 1.

Description: Recurrently differentially expressed genes in six CDX progression models, Related to Figure 2 and Supplementary Figure 2.

Recurrent significantly (padj < 0.05) upregulated (log2FC > 1) and downregulated (log2FC < -1) genes in six progression models vs. corresponding pre-treatment models. p values from negative binomial distribution, calculated by DESeq2.”
