# Supplementary material for: Soluble guanylate cyclase signalling mediates etoposide resistance in progressing small cell lung cancer
Source: Nat Commun. 2021 Nov 17;12:6652. doi: 10.1038/s41467-021-26823-6 (PMC8599617; doi:10.1038/s41467-021-26823-6)
Supplement: Supplementary file 5 — Reporting Summary [file 41467_2021_26823_MOESM5_ESM.pdf]

nature portfolio

Corresponding author(s): Caroline Dive

Last updated by author(s): Sep 20, 2021

Reporting Summary

Nature Portfolio wishes to improve the reproducibility of the work that we publish. This form provides structure for consistency and transparency in reporting. For further information on Nature Portfolio policies, see our Editorial Policies and the Editorial Policy Checklist.

Statistics

For all statistical analyses, confirm that the following items are present in the figure legend, table legend, main text, or Methods section.

n/a	Confirmed	
☐	☒	The exact sample size (*n*) for each experimental group/condition, given as a discrete number and unit of measurement
☐	☒	A statement on whether measurements were taken from distinct samples or whether the same sample was measured repeatedly
☐	☒	The statistical test(s) used AND whether they are one- or two-sided<br>*Only common tests should be described solely by name; describe more complex techniques in the Methods section.*
☐	☒	A description of all covariates tested
☐	☒	A description of any assumptions or corrections, such as tests of normality and adjustment for multiple comparisons
☐	☒	A full description of the statistical parameters including central tendency (e.g. means) or other basic estimates (e.g. regression coefficient) AND variation (e.g. standard deviation) or associated estimates of uncertainty (e.g. confidence intervals)
☐	☒	For null hypothesis testing, the test statistic (e.g. *F*, *t*, *r*) with confidence intervals, effect sizes, degrees of freedom and *P* value noted<br>*Give P values as exact values whenever suitable.*
☒	☐	For Bayesian analysis, information on the choice of priors and Markov chain Monte Carlo settings
☐	☒	For hierarchical and complex designs, identification of the appropriate level for tests and full reporting of outcomes
☐	☒	Estimates of effect sizes (e.g. Cohen's *d*, Pearson's *r*), indicating how they were calculated

Our web collection on statistics for biologists contains articles on many of the points above.

Software and code

Policy information about availability of computer code

Data collection	RT-qPCR: LightCycler® 96 software version 1.1.0.1320. IHC: HALO software v2.3. Image analysis for migration assays: Fiji version 1.52f. Western blot: BioRad software Image Lab 3.0.1. Cell viability assays: FLUOstar Omega plate reader software version 5.11 R3. Proliferation assays: IncuCyte ZOOM System Sartorius software version 2016A.
Data analysis	Code used to process data and generate figures in this study has been made available on GitLab (https://gitlab.com/cruk-mi/max-schenk-gucy/).<br>RNA-seq: RNA-seq data were aligned to Homo sapiens GRCh38 and Mouse GRCm38 assembly (Ensembl release 99) using STAR (version 2.6.1d) as part of the nf-core RNA-seq pipeline. These data were filtered using the bamcmp algorithm (version 2.0) to remove any mouse contamination reads. The counts matrix was generated using the filtered reads and the Rsubread package version 2.0.1. Differential expression analysis was conducted using DESeq2 (version 1.26.0) and model matrix accounted for paired testing. The log2 fold change values were shrunk via the apeglm algorithm (version 1.12.0) within DESeq2, and volcano plots generated using Enhanced Volcano (version 1.8.0). Gene set enrichment analysis was performed using generally applicable gene set enrichment for pathway analysis (GAGE) (version 2.36).<br>WES: Adapter sequences were removed from the reads using Cutadapt version 2.10. Alignment of WES data to Human reference genome GRCh38 and Mouse reference genome GRCm38 was performed using bwa-mem version 0.7.17. Reads originating from potential mouse contamination were removed using bamcmp (version 2.0), an algorithm to distinguish human and mouse reads. Picard version 2.19.0 and GATK tools version 4.1.7 was used for deduplication, realignment, and recalibration of aligned data. Mutect2 (version 4.1.7) was used to call somatic mutations (TP53 mutations were also present in the germline of patient 18 and 20 and RB1 mutations were present in the germline of patient 20) and VEP version 99 was used to annotate mutation calls. CN data was generated using CNVkit version 0.9.3. Cancer mutational signatures were identified from the variant calls using SigsPack in R (version 1.4.0).<br>RT-qPCR analysis was performed using LightCycler® 96 software version 1.1.0.1320. IHC analysis was performed using HALO software v2.3. Image analysis for migration assays was performed using Fiji (version 1.52f). Western blot images were analyzed using BioRad software Image Lab 3.0.1. sgRNAs were designed using CHOPCHOP v3. Cell viability assays were performed using FLUOstar Omega plate reader software version 5.11 R3. Proliferation assays were performed using IncuCyte ZOOM System Sartorius software version 2016A. Statistical analysis were

done using GraphPad Prism Version 8.2.0, R version 3.6.1, and SAS software version 9.4.

For manuscripts utilizing custom algorithms or software that are central to the research but not yet described in published literature, software must be made available to editors and reviewers. We strongly encourage code deposition in a community repository (e.g. GitHub). See the Nature Portfolio guidelines for submitting code & software for further information.

Data

Policy information about availability of data

All manuscripts must include a data availability statement. This statement should provide the following information, where applicable:

- Accession codes, unique identifiers, or web links for publicly available datasets
- A description of any restrictions on data availability
- For clinical datasets or third party data, please ensure that the statement adheres to our policy

The RNA-seq data generated in this study have been deposited in the EMBL-EBI ArrayExpress database under accession code E-MTAB-8465, titled 'RNA of Small Cell Lung Cancer Circulating Tumor Cells Derived Explants', (http://www.ebi.ac.uk/arrayexpress/experiments/E-MTAB-8465). The WES data generated in this study have been deposited under accession code E-MTAB-10880, titled 'Soluble Guanylate Cyclase Signalling Mediates Etoposide Resistance in Progressing Small Cell Lung Cancer', (https://www.ebi.ac.uk/arrayexpress/experiments/E-MTAB-10880/). The GenBank accession codes to Human reference genome GRCh38 is GCA_000001405.15 (https://www.ncbi.nlm.nih.gov/assembly/GCF_000001405.26/) and to Mouse reference genome GRCm38 GCA_000001635.2 (https://www.ncbi.nlm.nih.gov/assembly/GCF_000001635.20/). Source data are available as a source data file.

Field-specific reporting

Please select the one below that is the best fit for your research. If you are not sure, read the appropriate sections before making your selection.

☒ Life sciences ☐ Behavioural & social sciences ☐ Ecological, evolutionary & environmental sciences

For a reference copy of the document with all sections, see nature.com/documents/nr-reporting-summary-flat.pdf

Life sciences study design

All studies must disclose on these points even when the disclosure is negative.

Sample size	CDX models reported here are from successful engraftment of CDX from SCLC patients enrolled on study between 2012 and 2017. No sample size calculations were carried out as this is a hypothesis-generating study which seeks to evaluate the presence of diagnostic and novel SCLC biomarkers for future validation. Samples are continually collected and mice are monitored for tumour growth. p1 models were compared to donor patient biopsy histopathology analyses and by two trained pathologists, who verified that the resultant CDX matched the donor patient's disease using the clinically recognised diagnostic criteria. Subsequent passage then resulted in p3 tumours which were also compared back to the donor/p1 tumours and verified to be consistent with the initial features of that patient's disease. Transcriptomic analysis were carried out on 3 independent CDX tumours per model and found to be concordant between replicates. Sample size justification for in vivo studies was according to Murphy et al., Can Res 76(19) 5798-5809 (2016). In vitro studies as well as transcriptomic analysis were performed with 3 biological replicates. These sample sizes are typical for the in vitro experiments performed and were sufficient for statistical analysis.
Data exclusions	For in vivo efficacy studies with CDX17P sgNTA or CDX17P sgB1-2 cells, no animals were excluded throughout data analysis. For efficacy studies with L-NMMA, mice for which dosing schedules could not be completed due to a deterioration in clinical condition or body weight loss were excluded throughout data analysis.
Replication	Histopathology was carried out on whole tumor sections and western blotting was carried out out on lysates from 3 independent biological replicates per model. All cell culture assays as well as RT-qPCR was performed on 3 independent biological replicates and all attempts at replication were successful. RNAseq analyses were conducted on 3 independent biological replicates per model as described in relevant methods and legends.
Randomization	Mice were randomized deterministically at 150 – 250 mm3 by assignment to vehicle or chemotherapy treatment groups, by evenly distributing initial tumour volume sizes.
Blinding	During analysis all samples were given an unique identifier, but these were not blinded. For in vivo efficacy studies, group allocations were known during allocation, conducting, outcome assessment, and data analysis of experiment. In order to minimise potential confounding factors, tumour measurements were performed by members of the laboratory not directly involved in the project and animals belonging to different treatment groups were housed in separate cages.

Reporting for specific materials, systems and methods

We require information from authors about some types of materials, experimental systems and methods used in many studies. Here, indicate whether each material, system or method listed is relevant to your study. If you are not sure if a list item applies to your research, read the appropriate section before selecting a response.

Materials & experimental systems

n/a	Involved in the study
	☒ Antibodies
	☒ Eukaryotic cell lines
☒	Palaeontology and archaeology
	☒ Animals and other organisms
	☒ Human research participants
☒	Clinical data
☒	Dual use research of concern

Methods

n/a	Involved in the study
☒	ChIP-seq
☒	Flow cytometry
☒	MRI-based neuroimaging

Antibodies

Antibodies used

For western blotting: Rabbit recombinant anti-GUCY1B1 antibody 1:1,000 (Abcam, 154841), Rabbit phospho-VASP (Ser239) antibody 1:1,000 (Cell Signaling Technology, 3114, RRID:AB_2213396), Rabbit anti-VASP Antibody 1:5,000 (Bethyl Laboratories, A304-769A-M, RRID:AB_2782159), Rabbit anti-Synaptophysin antibody 1:20,000 (Abcam, ab32127, RRID:AB_2286949), Rabbit HES1 (D6P2U) mAb 1:500 (Cell Signaling Technology, 11988, RRID:AB_2728766), Rabbit GAPDH (14C10) mAb 1:5,000 (Cell Signaling Technology, 2118, RRID:AB_561053), Rabbit Notch1 Antibody 1:500 (Bethyl Laboratories, A301-895A, RRID:AB_1524102), Rabbit Histone H3 Antibody 1:5,000 (Cell Signaling Technology, 9715, RRID:AB_331563), Rabbit α-Tubulin Antibody 1:5,000 (Cell Signaling Technology, 2144, RRID:AB_2210548), Rabbit Vinculin Antibody 1:20,000 (Abcam, ab129002, RRID:AB_11144129), horseradish peroxidase-coupled secondary IgG 1:10,000 (Agilent, P044801-2, RRID:AB_2617138).
For IHC: Recombinant anti-GUCY1B1 antibody (Abcam, ab154841) at 6.76 µg/ml, pHH3 (Millipore, 06-570) at 0.4 µg/ml, and cCas3 (Cell Signaling Technology, 9661) at 0.2595 µg/ml , REST monoclonal antibody (CL0381) 1:150 (Thermo Fisher Scientific, MA5-24606, RRID:AB_2637221), SYP (Leica Biosystems, PA0299).
For Co-Immunofluorescence: GUCY1B1 primary antibody 1:100 (Abcam, 154841), HES1 primary antibody 1:100 (Cell Signalling Technologies,11988, RRID:AB_2728766), horseradish peroxidase-coupled secondary antibody (DAKO, K4003, RRID:AB_2630375).

Validation

All antibodies used in the study are commercially available and were validated by the manufacturers and protocols, specificity and validations can be found on the manufacturers' website:

For western blotting:
Rabbit recombinant anti-GUCY1B1 antibody: https://www.abcam.com/gucy1b3-antibody-epr8822-ab154841.html
Rabbit phospho-VASP (Ser239) antibody: https://www.cellsignal.co.uk/products/primary-antibodies/phospho-vasp-ser239-antibody/3114
Rabbit anti-VASP Antibody: https://www.bethyl.com/product/A304-769A/VASP+Antibody
Rabbit anti-Synaptophysin antibody: https://www.abcam.com/synaptophysin-antibody-ye269-ab32127.html
Rabbit HES1 (D6P2U) mAb: https://www.cellsignal.co.uk/products/primary-antibodies/hes1-d6p2u-rabbit-mab/11988
Rabbit GAPDH (14C10) mAb: https://www.cellsignal.co.uk/products/primary-antibodies/gapdh-14c10-rabbit-mab/2118
Rabbit Notch1 Antibody: https://www.bethyl.com/product/A301-895A/Notch1+Antibody
Rabbit Histone H3 Antibody: https://www.cellsignal.co.uk/products/primary-antibodies/histone-h3-antibody/9715
Rabbit α-Tubulin Antibody: https://www.cellsignal.co.uk/products/primary-antibodies/a-tubulin-antibody/2144
Rabbit Vinculin Antibody: https://www.abcam.com/vinculin-antibody-epr8185-ab129002.html
Horseradish peroxidase-coupled secondary IgG: https://www.agilent.com/en/product/immunohistochemistry/antibodies-controls/secondary-antibodies/goat-anti-rabbit-immunoglobulins-hrp-(affinity-isolated)-153244

For IHC:
Recombinant anti-GUCY1B1 antibody: https://www.abcam.com/gucy1b3-antibody-epr8822-ab154841.html
pHH3: https://www.merckmillipore.com/GB/en/product/Anti-phospho-Histone-H3-Ser10-Antibody-Mitosis-Marker,MM_NF-06-570?ReferrerURL=https%3A%2F%2Fapp.quartzy.com%2F&bd=1
cCas3: https://www.cellsignal.co.uk/products/primary-antibodies/cleaved-caspase-3-asp175-antibody/9661
REST monoclonal antibody (CL0381): https://www.thermofisher.com/antibody/product/REST-Antibody-clone-CL0381-Monoclonal/MA5-24606
SYP: https://shop.leicabiosystems.com/us/ihc-ish/ihc-primary-antibodies/pid-synaptophysin

For Co-Immunofluorescence:
Rabbit recombinant anti-GUCY1B1 antibody: https://www.abcam.com/gucy1b3-antibody-epr8822-ab154841.html
HES1 primary antibody: https://www.cellsignal.co.uk/products/primary-antibodies/hes1-d6p2u-rabbit-mab/11988
Horseradish peroxidase-coupled secondary antibody: https://www.agilent.com/store/en_US/Prod-K400311-2/K400311-2

Eukaryotic cell lines

Policy information about cell lines

Cell line source(s)

SCLC cell lines NCI-H196 (source: male; RRID:CVCL_1509), NCI-H524 (source: male; RRID:CVCL_1568), NCI-H1339 (source: female; RRID:CVCL_A472), NCI-H82 (source: male; RRID:CVCL_1591), NCI-H446 (source: male; RRID:CVCL_1562), and NCI-H1048 (source: female; RRID: CVCL_1453) were obtained from the American Type Culture Collection (ATCC).

Authentication	Cell lines were confirmed by STR profiling using the Promega PowerPlex 21 kit (Promega, DC8902) and analysed using genemapper5 software and an in-house database for comparisons/matching.
Mycoplasma contamination	Cell lines were tested negative for mycoplasma using a Venor®GeM-qEP Mycoplasma Detection Kit (Cambio, 11-9250) run on a QuantStudio 5 Real-Time PCR System (Thermo Fisher Scientific).
Commonly misidentified lines (See ICLAC register)	No commonly misidentified cell lines were used in this study.

Animals and other organisms

Policy information about studies involving animals; ARRIVE guidelines recommended for reporting animal research

Laboratory animals	For CDX model generation and studies testing efficacy of cisplatin/etoposide in sgNTA and sgB1-2 CDX17P, eight to 16 week-old female non-obese diabetic (NOD) severe combined immunodeficient (SCID) interleukin-2 receptor γ–deficient (NSG) mice (Charles River) were used. For L-NMMA efficacy studies, guided by a previous study testing L-NMMA and to increase tolerability to the compound (Granados-Principal et al., Breast Cancer Res, 2015), nine to eleven week-old female SCID Beige mice (Envigo) were used. Furthermore, all mice were drug/test naïve, did not undergo previous procedures and were housed in individually vented caging systems in a 12-h light/12-h dark environment and maintained at ambient temperature and humidity, and any cell implants and dosing was carried out in the morning on a laminar air flow bench and mice placed back in their home cages.
Wild animals	The study did not involve wild animals.
Field-collected samples	The study did not involve field-collected samples.
Ethics oversight	All procedures were performed in accordance with the Home Office Regulations (UK) and the UK Coordinating Committee on Cancer Research guidelines, by approved protocols (Home Office Project license nos. 40-3306/70-8252/P3ED48266), and the Cancer Research UK Manchester Institute Animal Welfare and Ethical Review Advisory Body.

Note that full information on the approval of the study protocol must also be provided in the manuscript.

Human research participants

Policy information about studies involving human research participants

Population characteristics	Data of patients contributing to CDX generation is summarized in Simpson, K. L. et al. A biobank of small cell lung cancer CDX models elucidates inter- and intratumoral phenotypic heterogeneity. Nature Cancer, doi:10.1038/s43018-020-0046-2 (2020). Patients were between 60 - 77 years old with extensive stage SCLC with metastasis to lymph nodes, liver, and bone. Patients received between 1-4 cycles of carboplatin and etoposide and survived between 2.7 - 10.5 months post diagnosis. Gender of patients contributing to paired CDX models: CDX3/3P: male, CDX8/8P: female, CDX17/17P: female, CDX18/18P: male, CDX20/20P male, CDX42/42P: male.
Recruitment	Material for the SCLC TMA was sourced through the approved Manchester Cancer Research Centre Biobank (MCRC), Project Reference 10_FIBL_01. Patients were identified in collaboration with the clinical accredited pathology department at Wythenshawe Hospital. A pathology review was conducted by a lead pathologist on those patients that had surgically resected and histologically confirmed SCLC (diagnosed 1993-2005) stored in the department which were initially used for diagnostic requirements but became surplus to diagnostic purpose. As these samples were an existing holding held prior to the HTA commencement date of 1st September 2006, it negated the requirement for patient consent. Specimens were processed to formalin fixed paraffin embedded blocks in line with pathology department approved SOPs. The TMAs were constructed in accordance to MCRC Biobank approved SOPs. Patients that contributed to CDX model generation were recruited by physician referral as follows: Patients diagnosed with SCLC were treated with standard of care chemotherapy, and survival times were in line with published averages. Although there was a bias for the generation of models from extensive stage disease patients, this represents a typical scenario for SCLC, since the majority of patients are diagnosed with extensive stage disease.
Ethics oversight	For those patient samples that contributed to the CDX generation, patients gave written informed consent to donate blood samples, pre, during and post treatment under the ethically approved ChemoRes (CHEMOtherapy RESistance) study, ethics reference - 07/H1014/96 approved by the North West Greater Manchester West Research Ethics Committee. The focus of this study is to investigate blood borne biomarkers for disease resistance in lung cancer patients.

Note that full information on the approval of the study protocol must also be provided in the manuscript.
